# 4,4'-Diisothiocyanostilbene-2,2'-disulfonic Acid (DIDS) Ameliorates Ischemia-Hypoxia-Induced White Matter Damage in Neonatal Rats through Inhibition of the Voltage-Gated Chloride Channel ClC-2

**DOI:** 10.3390/ijms160510457

**Published:** 2015-05-07

**Authors:** Baixiong Zhao, Hongyu Quan, Teng Ma, Yanping Tian, Qiyan Cai, Hongli Li

**Affiliations:** 1 Battalion 4 of Cadet Brigade, Third Military Medical University, Chongqing 400038, China; E-Mail: baixiong070@163.com; 2 Battalion 19 of Bioengineering, Third Military Medical University, Chongqing 400038, China; E-Mail: 18523505503@163.com; 3 Battalion 7 of Cadet Brigade, Third Military Medical University, Chongqing 400038, China; E-Mail: matt0119@163.com; 4 Department of Histology and Embryology, Third Military Medical University, 30# Gaotanyan St, Shapingba District, Chongqing 400038, China; E-Mails: tianyp1981@163.com (Y.T.); fengcai1112@126.com (Q.C.)

**Keywords:** DIDS, ischemia-hypoxia, Cl^−^ channel, oligodendrocyte, blocker, apoptosis

## Abstract

Chronic cerebral hypoperfusion is believed to cause white matter lesions (WMLs), leading to cognitive impairment. Previous studies have shown that inflammation and apoptosis of oligodendrocytes (OLs) are involved in the pathogenesis of WMLs, but effective treatments have not been studied. In this study, 4,4'-diisothiocyanostilbene-2,2'-disulfonic acid (DIDS), a chloride (Cl^−^) channel blocker, was injected into chronic cerebral ischemia-hypoxia rat models at different time points. Our results showed that DIDS significantly reduced the elevated mRNA levels and protein expression of chloride channel 2 (ClC-2) in neonatal rats induced by ischemia-hypoxia. Meanwhile, DIDS application significantly decreased the concentrations of reactive oxygen species (ROS); and the mRNA levels of inducible nitric oxide synthase (iNOS) and tumor necrosis factor-alpha TNF-α in neonatal rats with hypoxic-ischemic damage. Myelin staining was weaker in neonatal rats with hypoxic-ischemic damage compared to normal controls in corpus callosum and other white matter, which was ameliorated by DIDS. Furthermore, the elevated number of caspase-3 and neural/glial antigen 2 (NG-2) double-labeled positive cells was attenuated by DIDS after ischemia anoxic injury. Administration of DIDS soon after injury alleviated damage to OLs much more effectively in white matter. In conclusion, our study suggests that early application of DIDS after ischemia-hypoxia injury may partially protect developing OLs.

## 1. Introduction

The development of oligodendrocytes (OLs) involves multiple differentiation stages. Previous studies have reported that certain oligodendrocyte precursor cells at the late stage are very sensitive to hypoxic-ischemic injury [1]. These oligodendrocyte precursor cells are prone to injury and subsequently apoptosis, thereby impeding the maturation of myelin sheaths. This underlies the pathology of leukoencephalomalacia in the neonatal brain [2,3]. Previous studies have demonstrated that ischemia and hypoxia induce the excessive opening of chloride (Cl^−^) channels which results in altered physiological activities of cells [4]. The voltage-gated Cl^−^ channel 2 (ClC-2) is mainly involved in the regulation of cell volume and osmosis in developing cells. ClC-2 is especially closely associated with decreases in apoptotic volume during the early stages of cell injury [5]. Several studies have confirmed that Cl^−^ channels are significantly more open in myocardial cells after the reperfusion of ischemic tissues [6,7,8]. 4,4'-diisothiocyanostilbene-2,2'-disulfonic acid (DIDS), a Cl^−^ channel blocker, can prevent excessive opening of Cl^−^ channels and inhibit apoptosis-induced volume decreases (AVD) in myocardial cells [7,8]. DIDS also prevents cells from undergoing apoptosis, suggesting that Cl^−^ channel blockade may protect myocardial cells from ischemia-reperfusion injury. The protective mechanisms of DIDS in hypoxic-ischemic injury may play a role in the homeostasis of cellular antioxidant systems. The decreased capacity for scavenging oxygen free radicals, the toxicity of inflammatory factors [9], and the changes in metabolic pathways after hypoxic-ischemic injury may impede the generation of the antioxidants and deplete existing antioxidants which leads to OL damage and apoptosis. Therefore, DIDS is considered a likely protective agent for ischemia and hypoxia and has received extensive attention for its potential in clinical research. Similar findings have been reported in neurons of the central nervous system (CNS). These findings illustrate the important role of transporters and ion channels in neuronal injury under hypoxia [10]. However, the excessive opening of Cl^−^ channels and the inhibitory role of DIDS in OLs after cerebral white matter damage have not been fully elucidated. Therefore, we aimed to investigate the role of Cl^−^ channels in OLs after hypoxic-ischemic injury and the effects and related mechanisms of DIDS on OLs after hypoxic-ischemic damage. The pharmacological use of DIDS in hypoxic-ischemic injury may provide a foundation for a novel pharmacological treatment for neonatal ischemia and hypoxia in clinical settings.

## 2. Results

### 2.1. Chronic Ischemia and Hypoxia Induced ClC-2 Expression in the Cerebral White Matter

**Figure 1:**
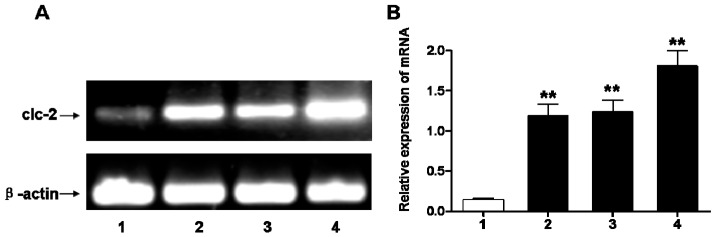
(**A**) ClC-2 mRNA expression changes in cerebral white matter after hypoxic-ischemic injury; (**B**) Relative expression of corresponding to ClC-2 mRNA compared to sham-operation group. Well 1: sham-operation group, Wells 2–4: 1, 3, 7 days after injury, respectively; Values represent means ± S.E.M. (*n* = 5). ** *p* < 0.01.

**Figure 2:**
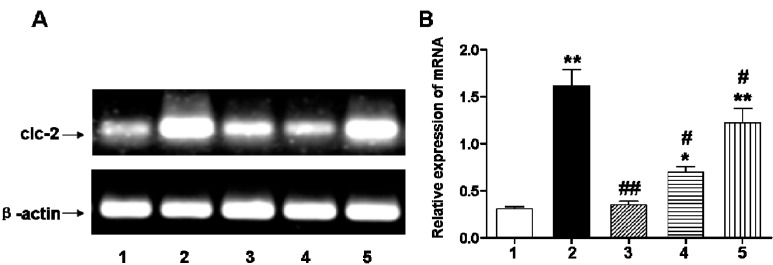
(**A**) ClC-2 mRNA relative expression changes in cerebral white matter before and after hypoxic-ischemic injury observed three days after injury; (**B**) Relative expression of corresponding to ClC-2 mRNA compared to sham-operation group. Well 1: sham-operated group; Well 2: ischemic and hypoxia group; Well 3: administration of DIDS at 1 h after injury; Well 4: administration of DIDS at 6 h after injury; Well 5: administration of DIDS at 2h before injury. Values presented as means ± S.E.M. (*n* = 5), * *p* < 0.05; ** *p* < 0.01 *vs.* sham-operated group; # *p* < 0.05; ## *p* < 0.01 *vs.* ischemic and hypoxia group.

To study the effect on ClC-2 protein expression in chronic hypoxic-ischemic injury, we used RT-PCR and Western blot analyses to quantify changes in mRNA and protein expression, respectively. The mRNA level of ClC-2 was significantly increased a day after the hypoxic-ischemic injury and continuously increased at days 3 and 7 after the injury (*p* < 0.01) compared to the sham-operated group (Figure 1). Administration of DIDS at 1 and 6 h after the hypoxic-ischemic injury significantly reduced the ClC-2 mRNA level (Figure 2, wells 3, 4) as compared to hypoxic-ischemia without DIDS treatment (well 2). While DIDS administration 1h after the injury showed the most significant effect on reducing ClC-2 mRNA level (*p* < 0.01), pre-administration of DIDS showed no effect on ClC-2 mRNA level as compared to the non-treatment group at 2 h after the hypoxic-ischemic injury (Figure 2, well 5), suggesting that the administration of DIDS at 1 h after the hypoxic-ischemic injury had the most impact on ClC-2 expression.

**Figure 3:**
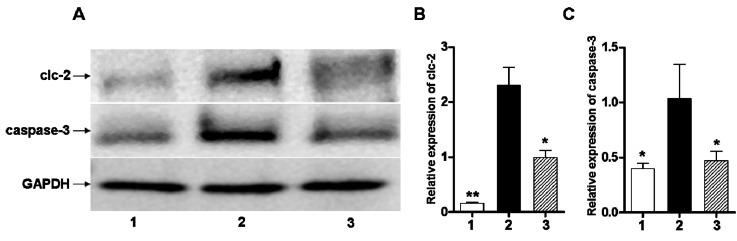
(**A**) ClC-2 protein relative expression changes, observed three days after injury, in cerebral white matter after hypoxic-ischemic injury; (**B**) Quantification of ClC-2 normalized to GAPDH expression; (**C**) Quantification of caspase-3 normalized to GAPDH expression. Well 1: sham-operated group. Well 2: ischemic and hypoxia group. Well 3: administration of DIDS at 1 h after injury. Values presented as means ± S.E.M. (*n* = 5), * *p* < 0.05; ** *p* < 0.01 *vs.* ischemic and hypoxia group.

The ClC-2 and caspase-3 protein expression levels, as determined by Western blot analysis, were significantly higher in the ischemic and hypoxia group than the sham-operation group (*p* < 0.01, *p* < 0.05) (Figure 3).

### 2.2. Early DIDS Administration during Hypoxic-Ischemic Injury Can Reduce the Concentration of Reactive Oxygen Species (ROS) and Inflammation

**Figure 4:**
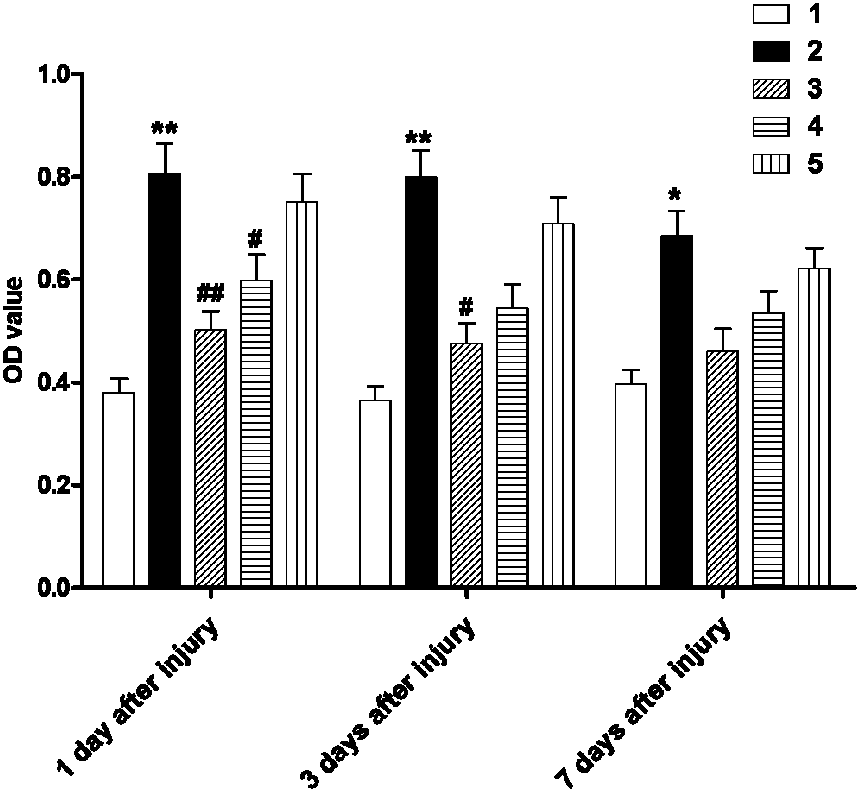
The effect of DIDS treatment on reactive oxygen species (ROS) in cerebral white matter of each group at different time points after hypoxic-ischemic injury. Well 1: sham-operation group. Well 2: ischemic and hypoxia group. Well 3: administration of DIDS at 1 h after injury. Well 4: administration of DIDS at 6 h after injury. Well 5: administration of DIDS at 2 h before injury. Values presented as means ± S.E.M. (*n* = 5), * *p* < 0.05; ** *p* < 0.01 *vs.* sham-operated group; # *p* < 0.05, ## *p* < 0.01 *vs.* ischemic and hypoxia group.

To determine whether the increased ClC-2 expression is involved in inflammation, we applied DIDS, a ClC-2 blocker during the early stages of hypoxic-ischemic injury and assessed changes in ROS concentration and inflammatory factors in the white matter. The ROS concentration significantly increased at day 1 after the hypoxic-ischemic injury and remained higher than the sham-operated group at postoperative day three and day seven (Figure 4; *p* < 0.01, *p* < 0.05, respectively). DIDS administration at 1 h after the hypoxic-ischemic injury significantly reduced the ROS concentration at day 1 as compared to the sham-operated group (*p* < 0.01). Similarly, DIDS administration at 6 h reduced the ROS concentration at day 1 after injury (*p* < 0.05). The effect of DIDS administration at 1 h after injury was sustained, in that ROS levels were reduced for three days (*p* < 0.05), whereas no significant effect at this later time point was found in other treatment groups (Figure 4).

**Figure 5:**
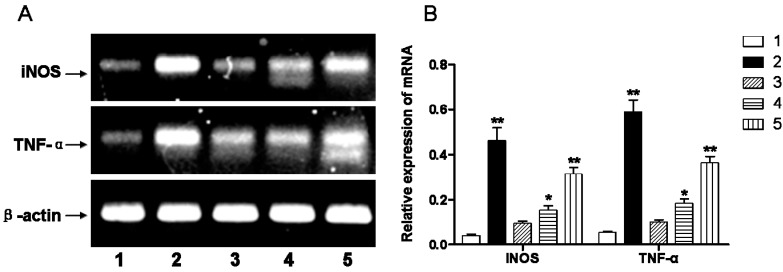
(**A**) Inflammatory factors iNOS and TNF-α mRNA relative expression changes, observed three days after injury, in cerebral white matter after hypoxic-ischemic injury; (**B**) Relative expression of corresponding to iNOS and TNF-α mRNA compared to sham-operation group. Well 1: sham-operated group. Well 2: ischemic and hypoxia group. Well 3: administration of DIDS at 1 h after injury. Well 4: administration of DIDS at 6 h after injury. Well 5: administration of DIDS at 2 h before injury. Values presented as means ± S.E.M. (*n* = 5), * *p* < 0.05; ** *p* < 0.01 *vs.* sham-operated group.

RT-PCR was used to quantify mRNA level of inflammatory cytokines (*i.e.*, inducible nitric oxide synthase, iNOS; tumor necrosis factor alpha, TNF-α). The mRNA levels of iNOS and TNF-α in the hypoxic-ischemic group were significantly higher than in the sham-operated group (*p* < 0.01). Administration of DIDS at 1 and 6 h after the hypoxic-ischemic injury reduced the mRNA levels of iNOS and TNF-α; while DIDS administration at 1 h demonstrated the most pronounced effect (Figure 5).

### 2.3. Early Administration of DIDS Reduced Demyelination

**Figure 6:**
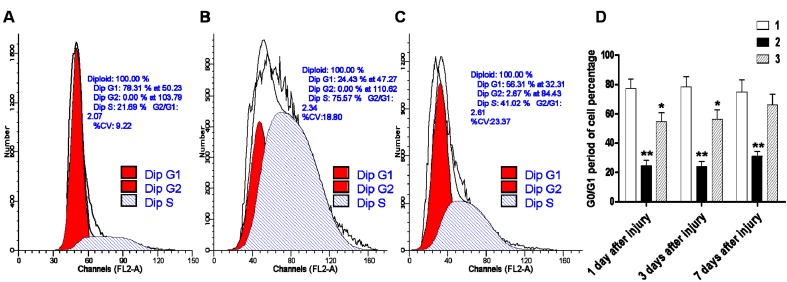
The effect of DIDS treatment on cell division cycles by fluid cytology after hypoxic-ischemic injury. (**A**) sham-operated group; (**B**) ischemic and hypoxia group; (**C**) administration of DIDS at 1 h after injury; (**D**) Relative expression of corresponding to G0/G1 period of cell percentage compared to sham-operation group. Well 1: sham-operated group. Well 2: ischemic and hypoxia group. Well 3: administration of DIDS at 1 h after injury. Values presented as means ± S.E.M. (*n* = 5), * *p* < 0.05; ** *p* < 0.01 *vs.* sham-operated group.

To determine whether the abnormal increase in ClC-2 expression after hypoxic-ischemic injury affects normal cell proliferation and differentiation, we examined the changes in the cell cycle kinetics by flow cytometry. The results showed that 24.43% of cells in the hypoxic-ischemic group were in the G0/G1 phase, and this percentage was significantly lower than the sham-operated group (78.31%) (*p* < 0.01). DIDS administration at 1 h after the hypoxic-ischemic injury increased the ratio of cells at the G0/G1 phase to 56.31%, and this percentage is significantly higher than the hypoxic-ischemic group without DIDS treatment but still lower than the sham-operated group (*p* < 0.05). Our data suggested that DIDS administration may block the abnormal expression of ClC-2 and promote restoration of the normal cell cycle ratio in the cerebral white matter (Figure 6).

**Figure 7:**
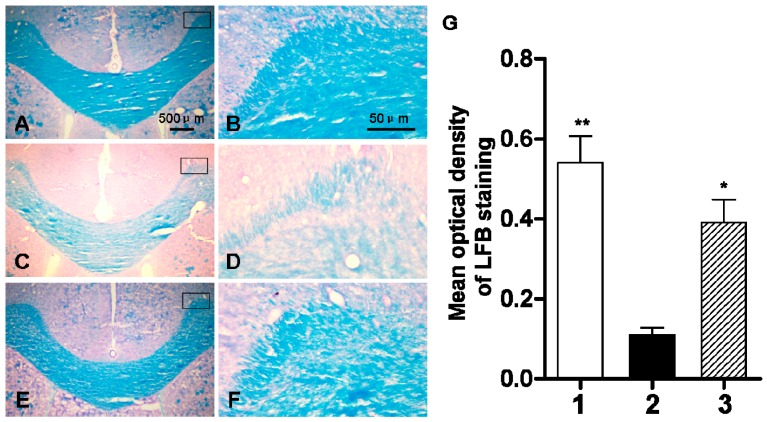
The changes of myelin development by myelin specific LFB staining three days after hypoxic-ischemic injury. (**A**,**B**) sham-operated group; (**C**,**D**) ischemic and hypoxia group; (**E**,**F**) administration of DIDS at 1 h after injury; (**G**) Quantification of LFB staining by optical density. Well 1: sham-operated group. Well 2: ischemic and hypoxia group. Well 3: administration of DIDS at 1 h after injury. Values presented as means ± S.E.M. (*n* = 5), * *p* < 0.05; ** *p* < 0.01 *vs.* ischemic and hypoxia group.

In order to observe the impact of abnormal ClC-2 expression in the cerebral white matter of rats during development, we examined the changes in the myelin sheath by Luxol fast blue (LFB) staining. In the sham-operated group, we observed a homogenous dark blue region in the cerebral white matter and a clear tissue margin outside the cerebral white matter (Figure 7). The staining intensity of LFB was lighter on day three of chronic ischemia and hypoxia with the mean optical density at 0.11 which is lower than that of the sham-operation group (0.54). The blue-stained region was significantly reduced with uneven staining intensity. DIDS administration at 1h after the hypoxic-ischemic injury resulted in a mean optical density value of 0.39, which was significantly higher than that of the hypoxic-ischemic group without treatment, but still lower than that of the sham-operated group (Figure 7).

### 2.4. Early Administration of DIDS Minimized the Apoptotic Ratio after Hypoxic-Ischemic Injury

**Figure 8:**
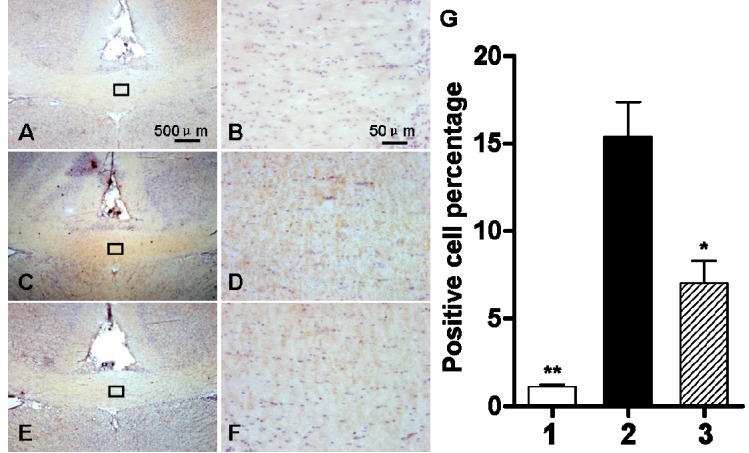
The effect of DIDS treatment on the number of ClC-2 positive cells three days after hypoxic-ischemic injury. (**A**,**B**) sham-operated group; (**C**,**D**): ischemic and hypoxia group; (**E**,**F**): administration of DIDS at 1h after injury; (**G**) Quantification of ClC-2 positive cells. Well 1: sham-operated group. Well 2: ischemic and hypoxia group. Well 3: administration of DIDS at 1 h after injury. Values presented as means ± S.E.M, (*n* = 5), * *p* < 0.05; ** *p* < 0.01 *vs.* ischemic and hypoxia group.

We examined ClC-2 protein expression in the apoptotic OLs in cerebral white matter after hypoxic-ischemic injury. As shown in Figure 8 immunohistochemical (IHC) staining revealed a significant increase in the number of ClC-2 positive cells in the corpus callosum of hypoxic-ischemic cerebral white matter (*p* < 0.01). The number of ClC-2 positive cells with DIDS administration at 1 h after the hypoxic-ischemic injury was significantly lower than that in the hypoxic-ischemic group without DIDS treatment (*p* < 0.05) (Figure 8).

**Figure 9:**
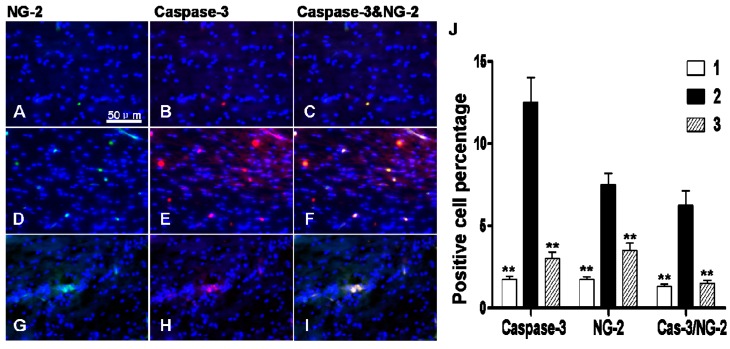
The effect of DIDS treatment on the number of caspase-3 and NG-2 positive cells 3 days after hypoxic-ischemic injury. (**A**–**C**) sham-operated group; (**D**–**F**) ischemic and hypoxia group; (**G**–**I**) administration of DIDS at 1 h after injury; (**J**) Quantification of caspase-3 and NG-2 positive cells. Well 1: sham-operation group. Well 2: ischemic and hypoxia group. Well 3: administration of DIDS at 1 h after injury. Values presented as means ± S.E.M. (*n* = 5), ** *p* < 0.01 *vs.* ischemic and hypoxia group.

Immunofluorescence staining demonstrated that there were more cells positive for caspase-3 and neural/glial antigen 2 (NG-2) antigen markers in the corpus callosum region of hypoxic-ischemic group (Figure 9D–F) than that of the sham-operated group (Figure 9A–C) (*p* < 0.01), suggesting an increase in apoptosis in OLs. DIDS administration at 1 h after chronic cerebral ischemia and hypoxia significantly decreased the activation of apoptotic pathways (*p* < 0.01) (Figure 9G–I).

## 3. Discussion

In this study, we analyzed the effects of DIDS treatment on hypoxic-ischemic injury in the expression of ClC-2 in the cerebral white matter. Chronic cerebral ischemia and hypoxia led to abnormal increase in ClC-2 expression. Early administration of a ClC-2 specific blocker, DIDS, significantly inhibited the overexpression of ClC-2 and reduced inflammation, ROS concentration, and the number of apoptotic OLs in the cerebral white matter. Furthermore, DIDS reduced demyelination and maintained the normal cell proliferation and differentiation cycles, suggesting that DIDS administration at the early stage of chronic hypoxic-ischemic injury can significantly reduce white matter pathology.

Cl^−^ channels transport Cl^−^ and other anions in the mammalian cells. This is involved in multiple complex physiological processes [11]. Recent studies have shown that Cl^−^ channel blockers are protective in myocardial ischemia-reperfusion injury. However, their potential for protection against hypoxia and ischemia has not been examined in other organs and in tissues of the nervous system. Hypoxia and ischemia are common injuries that can have severe consequences at the cellular level including cellular apoptosis, and OL precursors are sensitive to ischemia and hypoxia. Despite their own antioxidant system, OLs are highly vulnerable to oxidative stress because they are rich in membrane lipids and intracellular iron and have fewer antioxidant enzymes [12].

An elevated level of ROS is one of the major signs for antioxidant system imbalance. We found a significant elevation of ROS concentration in the white matter after hypoxic-ischemic injury [13,14]. Because elevated ROS could lead to structural and functional damage of the mitochondria, excess ROS triggers a caspase-dependent apoptosis in OLs [15].

Previous studies have demonstrated a protective effect of DIDS in hypoxic-ischemic injury by reducing the infiltration of inflammatory cells [16]. The secretion of toxic products and cytokines, in particular, the release of interleukin 1 (IL-1), TNF-α, and interferon-gamma (IFN-γ) can occur, which in turn could affect the REDOX system and impair the antioxidant system [17]. IFN-γ can directly damage OL precursors. Expression of IFN-γ receptors in the membrane of OL precursor cells and the release of TNF-α underlies IFN-γ-mediated damage of OL precursors [18]. This study demonstrates a significant decrease in the TNF-α and iNOS mRNA levels in white matter cells after hypoxic-ischemic injury and this decrease is induced by time-dependent administration of DIDS, supporting the hypothesis that inflammatory cytokines play an important role in hypoxic-ischemic injury.

Apoptosis is a serious consequence of an imbalance in the antioxidant system and thus could serve as an indicator of the level of oxidative damage. Moreover, apoptosis is the major pathological change after the oxidative damage in OLs [19]. Our data demonstrated a significant increase in the number of apoptotic OLs in cerebral white matter after hypoxic-ischemic injury, which further compromised myelin growth and development. Recently, Okada *et al.* [20] found that Cl^−^ channel blockers prevent cellular apoptosis by inhibiting the activation of early apoptotic cells, which also protects myocardial cells after hypoxic-ischemic injury. Apoptosis plays an important role in the pathological neuronal cell death during the development of the nervous system and in many neurodegenerative diseases such as cerebral stroke, traumatic brain injury, Alzheimer’s disease, Parkinson’s disease, and Huntington’s disease [21]. Pamenter *et al.* [22] reported that DIDS can protect the membrane integrity of neurons and improve cell survival. They also found that Cl^−^ channels are involved in *in vivo* ischemia and *in vitro* hypoxia/re-oxygenation-induced neuronal apoptosis in the hippocampal neurons of rats exhibiting nitric oxide-induced hippocampal neuronal apoptosis, suggesting that Cl^−^ channels have an important role in the neuronal apoptosis of the nervous system undergoing hypoxia and ischemia.

This study showed that hypoxic-ischemic injury induced the inflammation of cerebral white matter of neonatal rat, and this further promoted apoptosis of OLs and compromised the normal development of the myelin sheath. DIDS administration resulted in a significant protective effect in hypoxic-ischemic injury; DIDS administration at 1 h after hypoxic-ischemic injury substantially reduced the levels of intracellular ROS and inflammatory cytokines to below those observed in the hypoxic-ischemic group. These results were verified at the molecular, protein and cellular levels. Cellular protection through DIDS administration at 6 h after hypoxic-ischemic injury was diminished but still remained effective. Pre-administration of DIDS 2 h before injury showed no significant difference in its therapeutic effect among all hypoxic-ischemic groups.

## 4. Experimental Section

### 4.1. Animal Model

Clean, standard grade 4–5 day old newborn healthy Sprague-Dawley (SD) rats (both males and females), weighing 7 ± 2 g, were obtained from the Experimental Animal Center of The Third Military Medical University. The model of chronic cerebral ischemia and hypoxia in rats was established, with modifications, in accordance to protocols by Levine [23]. Rat necks were sterilized prior to operations and midline 5–8 mm incisions were made, ligature bilateral common carotid arteries, sent into neonatal rats anoxic crock after suturing skin, back to the mother’s side to continue breastfeeding after 2 h [24,25].

### 4.2. Experimental Animal Group

Rats were randomly divided into the following five groups: (1) sham-operated group; (2) hypoxic-ischemic damage group without DIDS treatment; (3) 1 h hypoxic-ischemic damage group + DIDS treatment; (4) 6 h hypoxic-ischemic damage group + DIDS treatment; (5) Pre-treatment with DIDS + 2 h anoxic-ischemic damage group. DIDS (Sigma-Aldrich, Saint Louis, MO, USA) was injected by intraperitoneal administration at a dose of 5 mg/kg.

### 4.3. Tissue Harvesting

The rats were sacrificed respectively at postoperative 1, 3 and 7 days. Brain white matter was harvested and tissue harvested for the following experiments: single cell suspensions for reactive oxygen species (ROS) detection and flow cytometry, protein extraction for Western blots, total RNA extraction for RT-PCR analysis, and frozen histological sections (20 µm) for immunohistochemistry (IHC).

### 4.4. RT-PCR

For RT-PCR, total RNA from white matter tissue was extracted with Trizol (Invitrogen, New York, NY, USA). Quality of extracted RNA was confirmed by spectrophotometer and agarose gel electrophoresis. 28 and 18 s bands were visible, and samples had no obvious degradation. The samples were stored at −80 °C until use. First chain cDNA synthesis was performed using kit following manufacturer’s instructions. PCR was done and primer sequences were as follows: ClC-2 forward sequence: 5'-AGA CAA TCC CTA CAC CCT TCA A-3', reverse sequence: 5'-TGT CGG TAG AAC ACC TTG TCA C-3'; TNF-α forward sequence: 5'-TGT GCC TCA GCC TCT TCT CAT-3', reverse sequence: 5'-ACC ACC AGT TGG TTG TCT TTG A-3'; iNOS forward sequence: 5'-TTG GAG CGA GTT GTG GAT TGT-3', reverse sequence: 5'-CGT TGT ACT CTG AGG GCT GAC A-3'; β-actin forward sequence: 5'-GAG ACC TTC AAC ACC CCA GCC-3', reverse sequence: 5'-TCG GGG GAT CGG AAC CGC TCA-3'. β-actin was used as a normalizing control.

### 4.5. Western Blot Analysis

Rats were decapitated and brain tissues were quickly removed from the skull. For total protein extracts, individual tissue samples were homogenized with ice-cold lysis buffer and protease inhibitors, and total protein quantified using Bradford assays; 100 µg was loaded per well and standard SDS gel electrophoresis and Western blotting techniques used. Immunoblots were incubated with primary antibodies against ClC-2 (1:500, Sigma-Aldrich, Saint Louis, MO, USA) or cleaved caspase-3 (1:500, Santa Cruz, Dallas, TX, USA) at 4 °C overnight. Horseradish peroxidase-conjugated secondary antibodies (1:5000, Santa Cruz) were used and immunoblots incubated at 37 °C for 4 h, followed by chemiluminescence detection for visualization. Protein bands were detected by the enhanced chemiluminescence method (ECL kit, Amersham, Pittsburgh, PA, USA) for 5 min. The β-actin protein was used as an internal control.

### 4.6. Immunohistochemistry Staining

For single-antibody immunostaining, frozen sections were rinsed three times in PBS, permeabilized and blocked with 10% goat serum in 0.1% (*w*/*v*) Triton X-100/PBS for 1 h at room temperature (RT). Then, sections were immunostained overnight at 4 °C using an antibody against ClC-2 (1:500, Sigma-Aldrich, USA). The following day, the sections were rinsed three times in PBS and incubated with a biotinylated anti-rabbit secondary antibody (Zhongshan, Beijing, China) at 37 °C for 4 h and DAB chromagen kit (Zhongshan, Beijing, China) used for detection.

For double-antibody immunostaining, sections were incubated with 5% normal goat serum and then incubated with rabbit anti-caspase-3 antibody (1:500, Sigma-Aldrich, USA) and mouse anti-NG-2 antibody (1:1000, Sigma-Aldrich, USA) overnight at 4 °C. Sections were incubated with a mixture of FITC- and TRITC-conjugated secondary antibodies (1:100, Abcam, Cambridge, MA, USA) at 37 °C for 4 h. Slides were counterstained with DAPI (1:800, Sigma-Aldrich, Saint Louis, MO, USA) after rinsing and being cover-slipped with fluorescence mounting medium (Dako, Copenhagen, Denmark). Tissues were visualized using an Olympus fluorescence microscope (Olympus, Tokyo, Japan), and digital images of sections acquired with a Charge-coupled Device camera.

### 4.7. Active Oxygen Concentration Detection

2',7'-dichlorofluorescin diacetate (DCFH-DA) 1 μL was added to 1 mL single cell suspensions, according to the reactive oxygen species (ROS) detection kit (Zhongshan, Beijing, China). The samples were incubated in the dark at 37 °C for 30 min. 1 μL rosup positive control was added to stimulate cells, and readings were taken 25 min later at 525 nm excitation wavelength in the spectrophotometer.

### 4.8. Flow Cytometry

OLs were collected from brain tissue, digested with 0.3% trypsin and single cells suspended in 0.01 mol/L PBS. Cells were washed twice and re-suspended in 100 μL 1 mg/mL RNase A at 37 °C for 30 min, and incubated with 400 μL 50 μg/mL propidium iodide (PI) and cells incubated for 10 min in the dark. Flow cytometry analysis was done by FACS-Calibur (BD Biosciences, Franklin Lakes, NJ, USA) instrument and CellQuest Software (Largo, FL, USA)used for data analysis.

### 4.9. Luxol Fast Blue (LFB) Staining

Sections were soaked in 0.1% LFB solution for myelin staining at 37 °C overnight. 95% ethanol was used to wash away excess dye solution, followed by 0.05% Li_2_CO_3_ solution, 70% ethanol, and distilled water until there was a sharp contrast from gray and white matter. The sections were immersed in 80%, 95%, 100% ethanol gradient dehydration washes for 10 min each, then mounted with neutral balsam. Sections were observed under a bright field microscope.

### 4.10. Statistical Analysis

All experiments were independently repeated in triplicates to ensure the reproducibility of the results, and representative results are shown. Quantitative data were presented as a means ± standard error of mean (SEM). Statistical significance was analyzed by performing one-way ANOVA followed by Dunnet’s post-hoc test. *p* ≤ 0.05 was considered statistically significant.
